# Effects of Climate on the Variation in Abundance of Three Tick Species in Illinois

**DOI:** 10.1093/jme/tjab189

**Published:** 2021-12-07

**Authors:** E A Bacon, H Kopsco, P Gronemeyer, N Mateus-Pinilla, R L Smith

**Affiliations:** College of Veterinary Medicine, University of Illinois, Urbana IL, USA

**Keywords:** abundance, temperature, precipitation

## Abstract

The range of ticks in North America has been steadily increasing likely, in part, due to climate change. Along with it, there has been a rise in cases of tick-borne disease. Among those medically important tick species of particular concern are *Ixodes scapularis* Say (Acari: Ixodidae), *Dermacentor variabilis* Say (Acari: Ixodidae), and *Amblyomma americanum Linneaus* (Acari: Ixodidae). The aim of this study was to determine if climate factors explain existing differences in abundance of the three aforementioned tick species between two climatically different regions of Illinois (Central and Southern), and if climate variables impact each species differently. We used both zero-inflated regression approaches and Bayesian network analyses to assess relationships among environmental variables and tick abundance. Results suggested that the maximum average temperature and total precipitation are associated with differential impact on species abundance and that this difference varied by region. Results also reinforced a differential level of resistance to desiccation among these tick species. Our findings help to further define risk periods of tick exposure for the general public, and reinforce the importance of responding to each tick species differently.

In North America, *Amblyomma americanum* Linneaus*, Dermacentor variabilis* Say, and *Ixodes scapularis* Say are major vectors for pathogens of humans, companion animals, and livestock (Jongegan and Uilenberg 2004, Lankester et al. 2007, Goddard and Varela-Stokes 2009, Diuk-Wasser et al. 2010, Dergousoff et al. 2013, Dahlgren et al. 2016, Eisen et al. 2016, Soucy et al. 2018). Together, these three tick species are implicated in the transmission of causal pathogens for a multitude of diseases including tularemia, ehrlichiosis, Lyme disease, spotted fever rickettsiosis, babesiosis, and anaplasmosis (Jongegan and Uilenberg 2004, Lankester et al. 2007, Goddard and Varela-Stokes 2009, Wasser et al. 2010, Brown et al. 2011, Dahlgren et al. 2016, Diuk-Eisen et al. 2016, Soucy et al. 2018). In recent decades, the increase in the geographical range of these species coincided with the rise in incidence of several tick-borne diseases including rickettsiosis and Lyme disease (Kollars et al. 1999, Diuk-Wasser et al. 2010, Dahlgren et al. 2016, Eisen et al. 2016, Hahn et al. 2016, Gilliam et al. 2020, Kugeler et al. 2021, Rosenberg et al. 2018, Alkishe et al. 2021). One proposed reason for altered tick phenology, geographic distribution, and disease transmission patterns is climate change (Ogden et al. 2014, Ogden and Lindsay 2016, Alkishe et al. 2021).

Numerous habitat suitability studies previously identified specific climate factors that influence tick activity and survival. Some of the most biologically relevant variables that restrict tick ranges and activity periods include maximum and minimum temperatures of hottest and coldest months (Sonenshine 1979, Vail and Smith 1998, Brownstein et al. 2003, Ogden et al. 2005, James et al. 2015, Hahn et al. 2016, Johnson et al. 2018, Minigan et al. 2018, Raghavan et al. 2019), relative humidity (Hair et al. 1975, Knulle and Rudolph 1982, Stafford 1994, Vail and Smith 1998, Rodgers et al. 2007, Berger et al. 2014) and vapor pressure (Brownstein et al. 2003, Diuk-Wasser et al. 2010, Springer et al. 2015, Hahn et al. 2016), and various measures of precipitation (Hahn et al. 2016, Johnson et al. 2018, Minigan et al. 2018, Raghavan et al. 2019).

Each of these biologically relevant variables, climate plays a differential role in determining the range and activity patterns of these three species and can directly impact their life histories (Ogden and Lindsay 2016).

The abiotic factors involving the two main vectors of Lyme disease-causing bacteria in North America, *I. scapularis* and *I. pacificus*, have been extensively studied. Researchers determined that as air temperature increases between 0 and 32°C, or as soil temperature increases, there is an associated decrease in nymphal developmental time for both *I. scapularis* and *I. pacificus* (Padgett and Lane 2001, Ogden et al. 2004). Additionally, the overall length of the entire life cycle of *I. scapularis* is positively correlated with decreasing temperature (Ogden et al. 2014). However, there are limited studies that examine the role of climate on life cycle timing (i.e., in what part of the year oviposition takes place) of ticks *across space.* One study by Lindsay and colleagues (1998) in Canada noted that female *I. scapularis* ticks tended to lay eggs two weeks sooner in habitats with warmer spring temperatures as compared to in habitats with cooler spring temperatures. However, no statistical analysis was performed to evaluate this observation (Lindsay et al. 1998). Moreover, in colder parts of North America, some *I. scapularis* ticks have been found to have a three to four-year-long life cycle as opposed to a two-year-long life cycle typical in many other parts of the continent (Yuval and Spielman 1990, Lindsay et al. 1998, Ogden et al. 2004, Eisen et al. 2016). These studies were also observational, and no statistical tests were performed to identify an association between differing climate and the duration of life cycle. Therefore, it remains uncertain whether differences in climate can help to explain existing differences in various temporal parameters of the life cycle of ticks within separate regions.

There is a further lack of research on the effects of climate on the life cycles of *A. americanum* and *D. variabilis*, and if they differ from one another. Studies suggest that climate may impact tick species in unique ways as a result of some species being more desiccation resistant than others (Schulze et al. 2002, Yoder et al. 2012). Evidence strongly demonstrates that *A. americanum* is more desiccation resistant than *I. scapularis* and *D. variabilis* (Schulze et al. 2001, Schulze et al. 2002, Yoder et al. 2012).

The aim of this study was to determine whether there is temporal or regional variation in the absolute abundance of *D. variabilis*, *A. americanum*, and *I. scapularis* collected on tick drags between Central and Southern Illinois. We hypothesized that regional climate differs between South and Central Illinois, and that these differences are predictive of existing temporal variation in tick abundance collected from tick drags. This prediction is supported by past research that found differences in life cycle timing and length of occurrence of blacklegged ticks across regions with various climates (Yuval and Spielman 1990, Lindsay et al. 1998, Eisen et al. 2003, Ogden et al. 2004, Ogden et al. 2005, Eisen et al. 2016). We additionally hypothesized that temperature, humidity, and precipitation will predict abundance of life stages, but will have differential effects based on the species of tick and the region. Furthermore, we expected that, regardless of region, increasing temperature combined with decreasing humidity and precipitation would be associated with lower adult abundance for *D. variabilis*, and both and nymphal and adult abundance for *I. scapularis* but not for *A. americanum.*

## Materials and Methods

### Data Collection

**Fig. 1.**
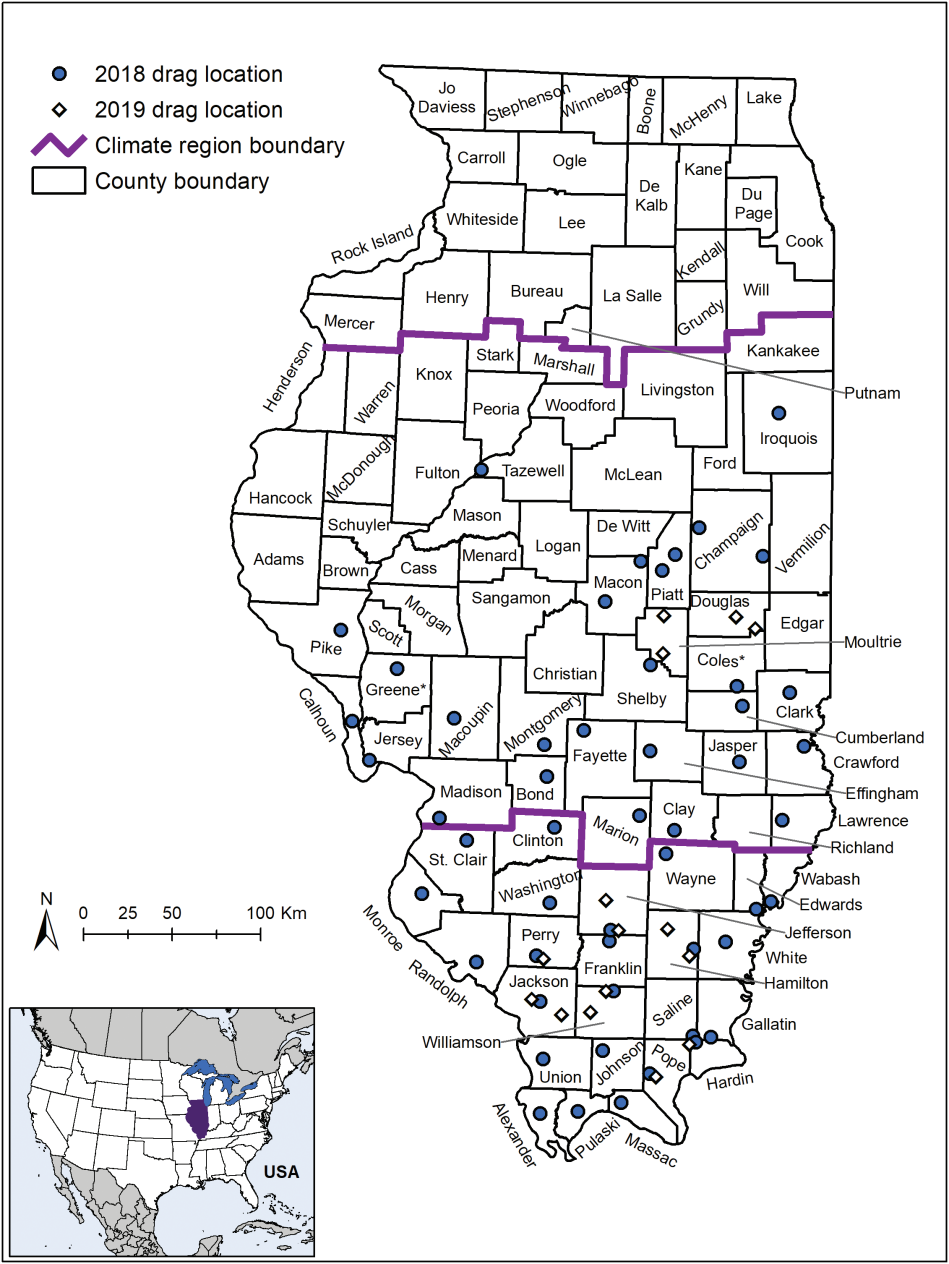
Map of the study area indicating the sites where the active surveillance was conducted during 2018 and 2019 in the central and southern regions of the state of Illinois.

Between May 2018 and November of 2019, ticks were collected via dragging eventsat sites in 24 central and 24 southern Illinois counties (Fig. 1; Table 1; Supp Table 1 [online only]) in the study area (Lyons et al. 2021). Researchers dragged 1 m^2^ white canvas flags with trailing ‘fingers’ along both sides of three established transects of 100 m, for a total of 600 m^2^ dragged per site, per visit (Lyons et al. 2021). Every 10–15 m, researchers stopped to check the drag cloth for any ticks. A total of three different habitat types were dragged at each site (i.e., forest, grassland, and ecotone). Active dragging in some locations (Champaign County) was conducted biweekly, while others were conducted monthly.

**Table 1.** Model corrected Akaike Information Criteria (AICc) by species, lifestage, and region for ticks in Central and Southern Illinois

Model	Dermacentor variabilis				Amblyomma americanum				Ixodes scapularis			
	Adult^*a*^		Nymph^*b*^		Adult^*a*^		Nymph^*a*^		Adult^*c*^		Nymph^*c*^	
	Central	South	Central	South	Central	South	Central	South	Central	South	Central	South
+T_max_	194.42	261.99	**13.20**	31.52	**85.62**	246.39	NA	362.70	37.68	25.81	NA	NA
+T_max_+VP_max_ +VP_min_	NA	**239.11**	NA	34.68	NA	233.96	147.26	358.50	47.26	NA	NA	NA
+VP_min_	191.96	269.00	13.90	32.57	91.02	258.96	154.67	382.67	46.21	33.82	47.04	41.63
+VP_max_+VP_min_	192.99	252.35	15.87	34.65	92.35	249.31	149.49	363.85	43.47	98.26	NA	NA
+T_max_+VP_min_	**182.07**	256.45	15.46	33.46	NA	250.94	NA	365.03	44.37	98.26	NA	NA
+Precip	201.74	268.54	13.92	**27.90**	90.88	236.76	163.70	378.69	34.71	37.01	**40.04**	**41.38**
+Precip+T_max_	191.32	246.51	15.08	28.97	90.00	**217.63**	160.43	359.78	**32.67**	100.28	NA	NA
+T_max_+DP	191.05	250.28	15.48	33.40	90.00	231.47	145.70	359.86	38.82	98.28	NA	NA
+T_max_+DP+VP_max_	187.67	245.34	NA	NA	NA	229.75	145.31	NA	40.50	NA	NA	NA
+T_max_+DP +VP_max_+VP_min_	NA	240.06	NA	NA	NA	231.52	NA	NA	64.49	NA	NA	NA
+DP	198.86	253.50	13.43	31.16	90.25	234.23	NA	360.52	39.16	25.91	NA	NA
+DP+VP_max_	189.94	247.51	15.64	33.31	90.16	233.50	146.60	357.84	39.10	98.33	NA	NA
+DP+VP_max_ +VP_min_	184.35	240.04	18.02	35.62	NA	230.14	150.52	359.37	42.73	NA	NA	NA
+VP_min_	191.40	250.50	13.60	32.51	92.45	244.17	151.86	358.84	34.65	**25.76**	NA	NA
+T_max_+VP_min_	190.22	246.94	NA	32.34	NA	235.91	**145.23**	**356.42**	40.93	99.07	NA	NA

Monthly climate variables were T_max_ (average daily maximum temperature), VP_max_ (average daily maximum vapor pressure deficit), VP_min_ (average daily minimum vapor pressure deficit), DP (average daily dew point), and Precip (total precipitation). NA: not applicable, model fit was not possible.

^*a*^AICc from zero-inflated negative binomial models.

^*b*^AICc from logistic regression models.

^*c*^AICc from zero-inflated Poisson models.

Adult and nymphal ticks were removed from the drag cloth with tweezers and placed into a vial with 90% ethanol and labeled with a transect ID that indicated the habitat type and transect number. Larvae were collected using a lint roller or tape and placed into a Ziploc bag labeled with the transect ID. Ticks were identified to species and life stage using dissecting microscopes. Standard morphological identification keys were used to identify to species (McIntosh 1932, Clifford et al. 1961, Cooney and Hays 1972, Keirans and Litwak 1989, Bouseman et al. 1990, Durden and Keirans 1996, Keirans and Durden 1998, Dubie et al. 2017).

Weather variables were obtained for each county from the Oregon State’s PRISM climate group (Daly et al. 2008). The PRISM Climate Group produces 4 km gridded weather data modeled using daily station data from several station networks. The larger station network, the Cooperative Observer Program (COOP) includes more than 100 stations in Illinois and had complete data for the time period of this study. The data used included total precipitation level per month, average maximum daily temperature per month, average minimum daily temperature per month, average mean daily temperature per month, average daily maximum vapor pressure deficit (VPD) per month, average daily minimum VPD per month, average daily dewpoint per month, and two measures of average daily relative humidity per month for each county sampled. These values were collected for both collection years: 2018 and 2019. The first measure of relative humidity was found using this formula: Relative Humidity = 100 * (EXP ((17.625 * TD)/(243.04 + TD))/EXP ((17.625 * T)/(243.04 + T))), where TD refers to dewpoint and T refers to temperature. The second measure was found using this formula: RH2 = 100 * POWER 112 − 0.1 * T) + TD)/112 + 0.9 * T)), 8)).

### Statistical Analysis

Data were cleaned and analyzed in RStudio version 3.6.1 (Core R Team, 2019) using the packages lubridate (Grolemund and Wickham 2011), readxl (Wickham et al. 2019), dplyr (Wickham et al. 2020), reshape2 (Wickham 2007), and stringr (Wickham 2019). Data cleaning involved calculating all weather variables for each county and season (see below), collating the 2018 and 2018 surveillance data with the weather data, and excluding larvae due to the small number collected. Code are available at https://github.com/rlsdvm/TickAbundanceModels.

The National Oceanic and Atmospheric Administration considers Illinois to consist of nine climate divisions (NOAA 2021). The divisions that were sampled (three through nine) included two climate sectors in Illinois: central and south (Fig. 1). The central region consisted of climate regions three through seven. The southern region consisted of climate regions eight and nine. For both collection years (2018 and 2019), data were grouped as late spring if the samples were collected in May, as summer if the samples were collected between June and August, and as fall if the data were collected between September and November. The data collected during late spring were excluded from the dataset as no counties in the southern region were sampled during this time.

We performed ANOVAs using the plotrix package (Lemon 2006) to assess relationships among the various climate variables and region. Any climate variables that varied significantly (alpha ≤ 0.05) by region were subsequently used in the regression models.

For both regions, either zero-inflated Poisson or zero-inflated negative binomial regression models were used based on the level of dispersion in the dataset (Venables and Ripley 2002, Jackman 2020, Fox and Weisberg 2019) to test the effects of the selected climate variables on nymphal abundance and adult abundance. Variables were considered significant at alpha ≤ 0.1 as this was an exploratory assessment. Multicollinearity between the climate variables was also checked for and models were altered based on results. The model with the lowest corrected AIC value (AICc) was considered the best fit model; AICc was used due to small sample sizes.

Bayesian network analysis was performed to capture the conditional relationships among climate (weather variables), region (central and south), and timeframe (late spring, summer, fall) for all tick abundance data. Bootstrapping was used to determine the relative strength of the predictive power of each independent variable on nymph and adult abundance for each of the three tick species. The bnlearn R package (Scutari 2010) was used for this analysis. An arc was defined as a statistical association between variables. Arcs going from total monthly precipitation to dewpoint, maximum and minimum vapor pressure deficit, and both measures of humidity were forced into the model based on known interdependent meteorological relationships (Eccel 2012). Arcs going into region or time frame were blocked from the model, as were arcs going from measures of tick abundance to any measures of climate, as these were not biologically possible.

## Results

**Fig. 2.**
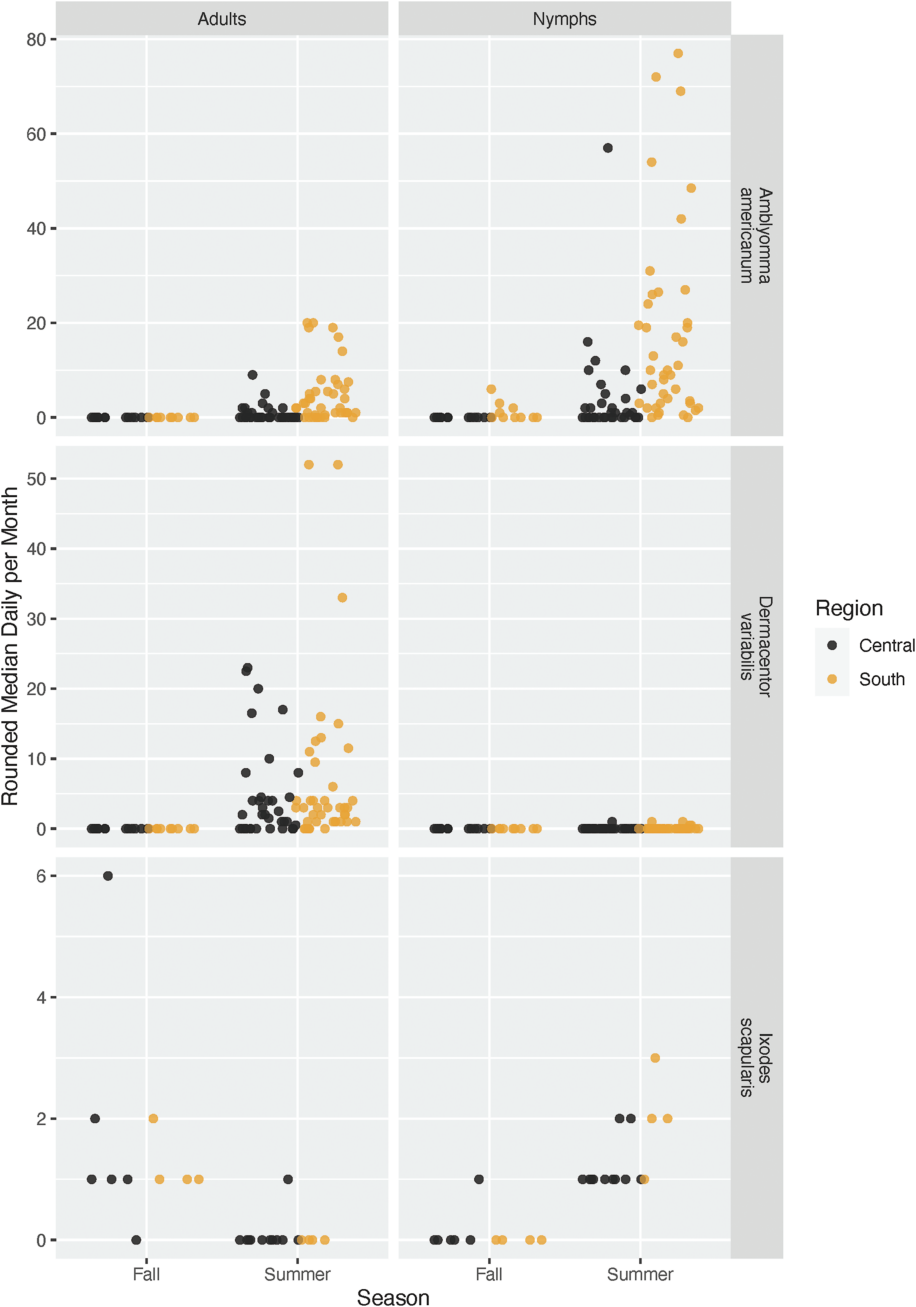
Observed number of ticks collected by region, timeframe, species, and life stage in Illinois in 2018 and 2019. To arrive at our tick counts, the median value of both adults and nymphs collected per day was found across collection sites per each county and time frame. Those values were then rounded to whole numbers.

Between May 2018 and November of 2019 there were 234 tick collection events during summer months (June–August), and 75 collection events during fall months (September–November), yielding a total of 37 *Ixodes scapularis* nymphs, 18 *I. scapularis* adults, 1121 *Amblyomma americanum* nymphs, 311 *A. americanum* adults, 5 *Dermacentor variabilis* nymphs, and 609 *D. variabilis* adults. Model results supported a strong seasonal effect across both regions, with most species and life stages being collected in the summer rather than the fall (Fig. 2). The only exception is *I. scapularis* adults, which were only found in the fall (since ticks were not collected in early spring).

Supp Figure 1 (online only) shows the distributions of the different variables collected and their pairwise correlation. Many of the climate variables were strongly correlated amongst each other, except for monthly total precipitation. Many of the tick observations were also correlated amongst each other, with the exceptions being *I. scapularis* adults (which, as noted, were collected in a different season than the other ticks) and *D. variabilis* nymphs, for which no collection event ever collected more than one.

### Regression Modeling of Environmental Correlates

The corrected AIC value of each model as fit to each region, species, and life stage is shown in Table 1, and the coefficients of the best-fit models for each region, species, and life stage are shown in Table 2. The monthly average of the daily maximum temperature, *T*_*max*_, was included in 8 of the 12 best fit models, with two of those models including only *T*_*max*_. The exceptions to this were for *I. scapularis* adults in the Southern region, which included the minimum daily vapor pressure, which is highly and positively correlated with *T*_*max*_ (Suppl Fig. 1 [online only]), and *I. scapularis* nymphs in both regions and *D. variabilis* nymphs in the Southern region, where the best fit model included only precipitation. In almost all cases, higher *T*_*max*_ was associated with a lower probability of finding ticks, e.g., differentiating where ticks were and were not found, but not necessarily a lower abundance where ticks were found. The exceptions to this relationship were *D. variabilis* and *A. americanum* nymphs in the Central region, which were positively associated with *T*_*max*_.

**Table 2.** Model coefficients for the best-fit models for tick abundance in Illinois by region, species, and lifestage using generalized linear models

Species	Life-stage	Region	Intercept		T_max_		DP		VP_max_		VP_min_		Precip	
			Count	Zeros	Count	Zeros	Count	Zeros	Count	Zeros	Count	Zeros	Count	Zeros
*Dermacentor variabilis*	Adult^*a*^	Central	−0.01	208.31	0.09	−6.96					−0.87	−11.19		
		South	27.96	41.57	−1.49	−7.14			1.12	9.42	−4.23	−23.87		
	Nymph^*b*^	Central		−23.94		0.68								
		South		−6.47										0.02
*Amblyomma americanum*	Adult^*a*^	Central	−1.89	303.31	0.07	−10.47								
		South	2.96	441.83	−0.08	−12.87							0.01	−0.62
	Nymph^*a*^	Central	−19.65	2.02	1.59	2.04			−1.32	−3.26				
		South	−6.89	75.29	0.65	−8.29			−0.51	7.42				
*Ixodes scapularis*	Adult^*c*^	Central	60.72	16.92	0.41	−0.53							−0.90	−0.03
		South	5.74	43.30			33.41	−5.78	−24.59	1.95	57.05	6.86		
	Nymph^*c*^	Central	0.03	163.43									0.00	−1.77
		South	1.36	−1.65									−0.01	0.01

Monthly climate variables were T_max_ (average daily maximum temperature), DP (average daily dew point), VP_max_ (average daily maximum vapor pressure deficit), VP_min_ (average daily minimum vapor pressure deficit), and Precip (total precipitation).

^*a*^Zero-inflated negative binomial models.

^*b*^Logistic regression models.

^*c*^Zero-inflated Poisson models.

The monthly average of the dew point was negatively associated with the probability of collecting *I. scapularis* adults in the Southern region. However, dew point was not included in any other best-fit model, likely due to its high correlation with *T*_*max*_.

Monthly total precipitation was included in the best-fit models for *D. variabilis* and *I. scapularis* nymphs and *A. americanum* adults in the Southern region only, and with *I. scapularis* adults in both regions. For these species and stages, increased precipitation was associated with decreased presence, and a very small increase in abundance were present. However, monthly total precipitation was positively correlated with presence of nymphal *D. variabilis* and *I. scapularis*.

### Bayesian Network Analysis to Predict TICK abundance

**Fig. 3.**
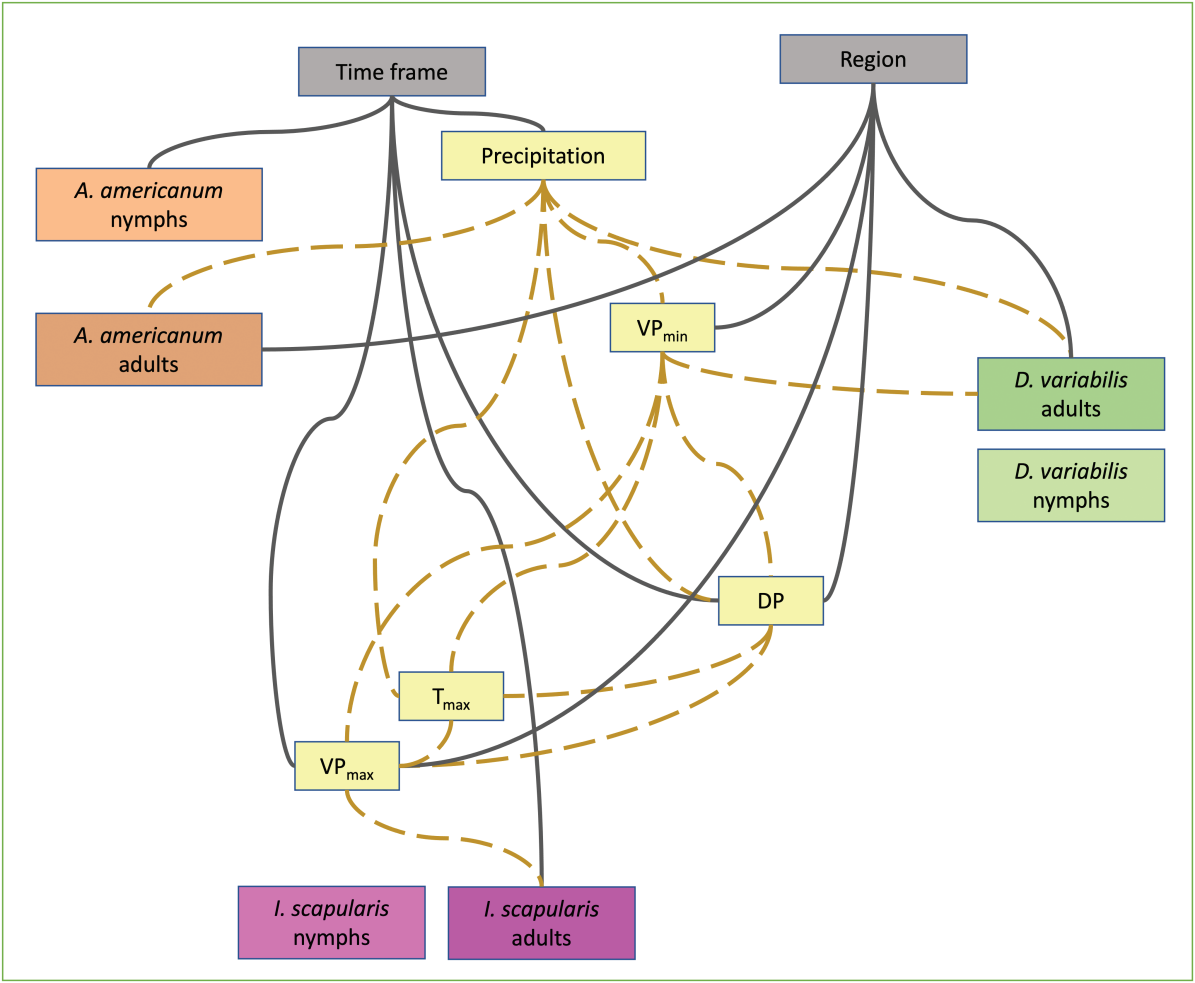
Best-fit Bayesian network model for observed tick abundance and climate in Illinois in 2018 and 2019. Gray solid lines indicate relationships with temporal and regional differences. Yellow dashed lines indicate relationships with weather variables.

Figure 3 depicts the final fitted Bayesian Network model, using 0.4 as the threshold value for inclusion in the model, and Table 3 shows the coefficients from the final Bayesian Network model fitted to the full dataset. For most species, only monthly total precipitation or a climate variable with a downstream effect from precipitation were strong predictors of abundance. Region was not associated with nymphal abundance of any species, nor with *I. scapularis* adults, while season was only associated with *A. americanum* nymphs and *I. scapularis* adults. However, both region and season were strongly associated with climate variables. Both *I. scapularis* and *D. variabilis* nymphs were found not to have strong enough relationships to be included in the final networks, likely due to the more limited number of observations available for these life stages.

**Table 3.** Coefficients for the best-fit Bayesian network model for tick abundance in Illinois by region, species, and lifestage using generalized linear models

Observed variable	Region	Intercept		Precipitation		T_max_		DP		VP_max_		VP_min_	
		Fall	Summer	Fall	Summer	Fall	Summer	Fall	Summer	Fall	Summer	Fall	Summer
Precip	All	92.5	152										
T_max_	All	8.57	6.02	0.0084	0.0056			1.33	1.16			−1.4	1.49
DP	Central	−5.01	19.4	0.028	−7.83E-05							16.8	−0.93
	South	16.4	19.9	−0.051	−0.0051							2.24	0.40
VP_max_	Central	0.053	−25.5	0.017	0.00078	0.415	2.28	0.419	−1.32			−0.55	0.81
	South	−15.9	−19.7	−0.0044	−0.00031	2.03	1.68	−1.29	−0.653			−1.02	1.06
VP_min_	Central	0.80		0.00084									
	South	0.77		−0.00050									
*Ixodes scapularis* Nymphs	All	0.22											
*Dermacentor Variabilis* Nymphs	All	0.050											
*Amblyomma Americanum* Nymphs	All	0.48	11.4										
*Ixodes scapularis* Adults	All	1.84	0.27							−0.077	−0.013		
*Dermacentor Variabilis* Adults	Central	−3.4		0.041								1.48	
	South	15.1		0.028								−19.44	
*Ixodes scapularis* Adults	Central	−0.28		0.0065									
	South	−0.41		0.031									

Monthly climate variables were T_max_ (average daily maximum temperature), DP (average daily dew point), VP_max_ (average daily maximum vapor pressure deficit), VP_min_ (average daily minimum vapor pressure deficit), and Precip (total precipitation).

Climate variables were interrelated, and those relationships were different between regions and time frames. In contrast, almost all tick variables were found to increase with increasing monthly total precipitation, although the size of the effect varied between regions. The rounded median daily adults per month per sampling for *D. variabilis* was found to be associated with minimum vapor pressure, but this relationship was weakly positive in the Central region and strongly negative in the Southern region.

## Discussion

Results suggest that climate does appear to have a differential effect by region within Illinois on prevalence and abundance of ticks across life stage and species. Although counts of *Dermacentor variabilis* nymphs were too low to analyze abundance, all other species and life stage combinations showed that both prevalence and abundance could be affected by climate variables differently in Central Illinois than in Southern Illinois. These findings support the literature on *Ixodes scapularis* (Hair et al. 1975, Brown et al. 1979, Stafford III 1994, Brownstein et al. 2003, James et al. 2015, Diuk-Wasser et al. 2010, Springer et al. 2015, Hahn et al. 2016, Johnson et al. 2018, Minigan et al. 2018, Raghavan et al. 2019) and expand the finding to the other important vector tick species in Illinois.

Using zero-inflated linear modeling approaches, the most consistent finding was related to the monthly averages of daily maximum temperature. Most observations that successfully collected ticks were in summer, with only *Ixodes scapularis* adults and small numbers of *I. scapularis* and *A. americanum* nymphs collected in the fall. Temperature was significantly higher during the summer collection dates in Southern Illinois, creating a potential risk of desiccation. In fact, only *Dermacentor variabilis* and *A. americanum* nymphs showed a positive relationship with temperature, and only in Central Illinois where temperatures were generally lower. This suggests that, to an extent, the nymphs of these two tick species may be more resistant to desiccation than the nymphs of *I. scapularis* and are able to quest during warmer temperatures. This is in agreement with past literature that found that *A. americanum* ticks were collected at greater frequency during warmer temperatures, and in less humid microenvironments, than *I. scapularis* ticks (Schulze et al. 2001, Schulze et al. 2002). Interestingly, that study examined tick adults while we found this result for nymphs (Schulze et al. 2001). These results for *D. variabilis* nymphs should be confirmed using host-based surveillance methods, however, due to the low reliability of drags for these ticks.

Our Bayesian network analysis found that precipitation was the most consistent predictor of tick abundance when controlling for the interrelationships of the climate variables, and that the role of precipitation changed by region. This is in agreeance with existing literature that found that higher precipitation levels in the warmest quarter of the year predict higher levels of habitat suitability for *I. scapularis* (Hahn et al. 2016) and that precipitation has a strong influence on *D. variabilis* (Minigan et al. 2018). In our linear modeling approach, by contrast, precipitation was only included in best-fit models in the southern region for *A. americanum* and *D. variabilis* and was negatively correlated with the probability of *I. scapularis* presence. However, the Bayesian network showed that precipitation was closely related to other climate variables, including temperature, and that all climate variables were affected by region and season. It is likely that the potential for multicollinearity among climate variables is responsible for the difference in model fitting between the two approaches. In most linear best-fit models that did not include precipitation, either vapor pressure deficit or dewpoint was included. Monthly total precipitation directly strongly influences both maximum VPD and dewpoint, resulting in an indirect effect of precipitation within those models as well. Previously, it has been suggested that precipitation is associated with tick abundance due to the impact it has on humidity, a factor that has been demonstrated to decrease the likelihood of *Ixodes* tick desiccation (Hahn et al. 2016, Hacker et al. 2021). Our results support these findings.

One caveat to this work is the limited surveillance data available for fitting these models. Unlike areas with more established tick surveillance (Diuk-Wasser et al. 2006), the state of Illinois has not been subject to repeated, in-depth tick dragging. Thus, these results must be interpreted with caution, as additional surveillance may provide a clearer picture of the regional tick abundance. However, the results presented here can guide targeted surveillance efforts towards areas and times most likely to be associated with tick activity. As many areas of the Midwest U.S. are still in the process of establishing the presence of vector ticks of concern, this could be useful to maximize the efficiency of resource-limited vector surveillance programs (Gilliam et al.). These results are also limited by the use of dragging methods alone; in particular, surveillance of *D. variabilis* nymphs would be improved by addition of host sampling, which has not been widely employed in Illinois.

Our ability to detect association with climate variables is limited because we measured climate on a broad scale as opposed to at the microhabitat level. Ticks are likely be more influenced by microclimate, such as relative humidity in leaf litter, as opposed to macroclimate, such as relative humidity in the total surrounding area (Bertrand and Wilson 1996, Vail and Smith 1998, Lindsay et al. 1998, James et al. 2015, Berger et al. 2014). We did not control for the influence of habitat type and it is possible that this could be the main driver of adult abundance differences between region (Hahn et al. 2016, Gilliam et al. 2018, Soucy et al. 2018, Wojan et al. 2021). Future studies should measure the relative importance of microclimate and macroclimate in tick abundance, controlling for habitat type. In addition, we were unable to account for colonization time by species, as due to the lack of a state-wide surveillance prior to 2018, this has not been well recorded in Illinois. It is assumed that *I. scapularis* has invaded from the north, meaning that it could still be expanding into the southern region, while *A. americanum* is believed to have invaded from the south, meaning that it could still be expanding into the southern region. Recent work by Wojan et al. (2021) found similar regional differences in tick abundance in Indiana, demonstrating the northward expansion of *A. americanum* and southward movement of *I. scapularis*. The extent to which each of the studied climate factors contributes to the permissive environment for continued colonization of the state requires further study. It would be of interest to repeat this analysis in future years to identify changes in detected presence and abundance of ticks due to expanding ranges and in association with colonization patterns.

This research indicates that the abundance of tick species is differentially impacted by climate across the central and southern regions of Illinois. This will be important for predicting risk periods for ticks in the different regions and communicating that risk to the public.

## Supplementary Data

Supplementary data are available at *Journal of Medical Entomology* online.

Supplemental Figure 1: Correlation among all variables used in models of climate effects on tick abundance in Illinois in 2018 and 2019

tjab189_suppl_Supplementary_Figure_S1

tjab189_suppl_Supplementary_Table_S1
