# Supplementary figures and images for: Effects of Climate on the Variation in Abundance of Three Tick Species in Illinois
Source: J Med Entomol. 2021 Dec 7;59(2):700–9. doi: 10.1093/jme/tjab189 (PMC8924963; doi:10.1093/jme/tjab189)

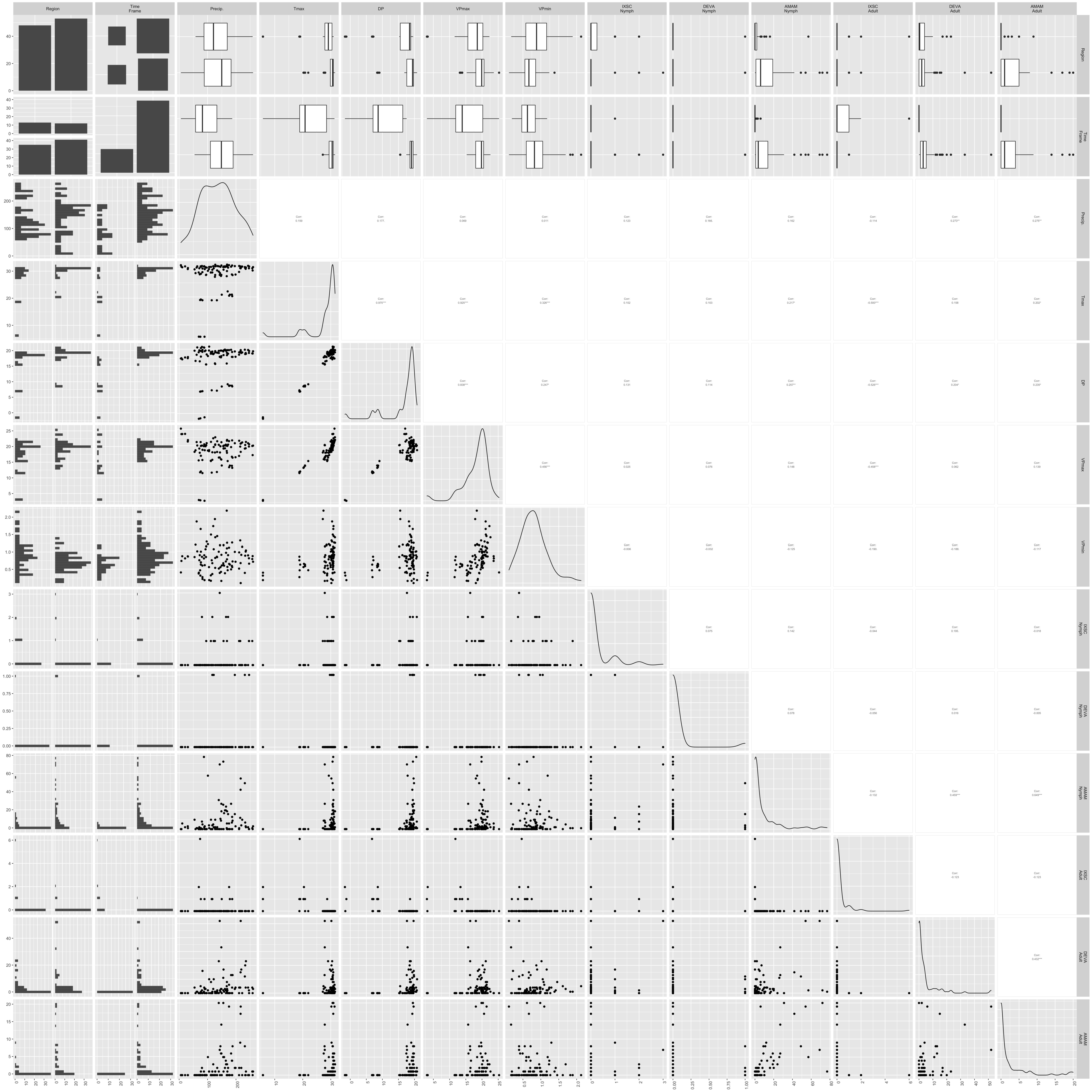

Supplement: tjab189_suppl_Supplementary_Figure_S1 [file tjab189_suppl_supplementary_figure_s1.jpeg]
