# Supplementary material for: Effects of Climate on the Variation in Abundance of Three Tick Species in Illinois
Source: J Med Entomol. 2021 Dec 7;59(2):700–9. doi: 10.1093/jme/tjab189 (PMC8924963; doi:10.1093/jme/tjab189)
Supplement: tjab189_suppl_Supplementary_Table_S1 [file tjab189_suppl_supplementary_table_s1.docx]

Supplemental Table 1: Data by county on surveillance effort and ticks collected

| County | Number of drags | *Amblyomma americanum* | | *Dermacentor variabilis* | | *Ixodes scapularis* | | Region |
| --- | --- | --- | --- | --- | --- | --- | --- | --- |
|  |  | Adult | Nymph | Adult | Nymph | Adult | Nymph |  |
| Alexander | 1 | 3 | 19 | 3 | 0 | 0 | 0 | Southern |
| Bond | 1 | 0 | 6 | 8 | 0 | 0 | 0 | Central |
| Calhoun | 1 | 3 | 5 | 3 | 0 | 0 | 0 | Central |
| Champaign | 24 | 0 | 0 | 1 | 0 | 1 | 3 | Central |
| Clark | 1 | 0 | 0 | 1 | 0 | 0 | 0 | Central |
| Clay | 1 | 2 | 1 | 4 | 0 | 0 | 0 | Central |
| Clinton | 1 | 4 | 13 | 4 | 0 | 0 | 0 | Southern |
| Coles | 1 | 0 | 0 | 0 | 0 | 0 | 0 | Central |
| Crawford | 1 | 0 | 0 | 2 | 0 | 0 | 1 | Central |
| Cumberland | 1 | 0 | 1 | 4 | 0 | 0 | 1 | Central |
| Douglas | 17 | 2 | 1 | 22 | 0 | 2 | 1 | Central |
| Edwards | 1 | 0 | 0 | 0 | 1 | 0 | 1 | Southern |
| Effingham | 1 | 0 | 2 | 23 | 0 | 0 | 0 | Central |
| Fayette | 1 | 2 | 4 | 17 | 0 | 0 | 0 | Central |
| Franklin | 1 | 4 | 20 | 2 | 0 | 0 | 0 | Southern |
| Gallatin | 1 | 11 | 53 | 25 | 0 | 0 | 0 | Southern |
| Greene | 1 | 0 | 0 | 0 | 0 | 0 | 0 | Central |
| Hamilton | 10 | 40 | 125 | 64 | 0 | 1 | 3 | Southern |
| Hardin | 1 | 19 | 54 | 52 | 0 | 0 | 0 | Southern |
| Iroquois | 1 | 0 | 0 | 0 | 0 | 0 | 1 | Central |
| Jackson | 10 | 36 | 47 | 8 | 0 | 1 | 2 | Southern |
| Jasper | 1 | 0 | 3 | 4 | 0 | 0 | 0 | Central |
| Jefferson | 10 | 31 | 92 | 22 | 1 | 1 | 0 | Southern |
| Jersey | 1 | 0 | 10 | 8 | 0 | 0 | 0 | Central |
| Johnson | 1 | 5 | 17 | 1 | 0 | 0 | 0 | Southern |
| Lawrence | 1 | 9 | 12 | 4 | 0 | 0 | 1 | Central |
| Macon | 24 | 1 | 1 | 88 | 0 | 3 | 4 | Central |
| Macoupin | 1 | 0 | 2 | 10 | 1 | 0 | 1 | Central |
| Madison | 1 | 2 | 2 | 2 | 0 | 0 | 0 | Central |
| Marion | 1 | 0 | 10 | 0 | 0 | 0 | 0 | Central |
| Massac | 1 | 0 | 2 | 4 | 0 | 0 | 0 | Southern |
| Monroe | 1 | 1 | 2 | 1 | 0 | 0 | 0 | Southern |
| Montgomery | 1 | 2 | 16 | 0 | 0 | 0 | 2 | Central |
| Moultrie | 20 | 1 | 9 | 32 | 0 | 0 | 0 | Central |
| Perry | 10 | 11 | 45 | 18 | 0 | 2 | 2 | Southern |
| Piatt | 25 | 0 | 0 | 15 | 0 | 7 | 14 | Central |
| Pike | 1 | 0 | 1 | 1 | 0 | 0 | 0 | Central |
| Pope | 7 | 30 | 130 | 91 | 1 | 0 | 0 | Southern |
| Pulaski | 1 | 19 | 68 | 6 | 0 | 0 | 0 | Southern |
| Randolph | 1 | 1 | 10 | 1 | 0 | 0 | 0 | Southern |
| Saline | 1 | 4 | 39 | 6 | 0 | 0 | 0 | Southern |
| Shelby | 1 | 0 | 7 | 20 | 0 | 0 | 0 | Central |
| StClair | 1 | 0 | 1 | 1 | 0 | 0 | 0 | Southern |
| Tazewell | 1 | 5 | 57 | 2 | 0 | 0 | 0 | Central |
| Union | 1 | 6 | 19 | 3 | 0 | 0 | 0 | Southern |
| Wabash | 1 | 1 | 3 | 3 | 0 | 0 | 0 | Southern |
| Washington | 1 | 2 | 16 | 3 | 1 | 0 | 0 | Southern |
| Wayne | 1 | 17 | 42 | 15 | 0 | 0 | 0 | Southern |
| White | 1 | 1 | 0 | 2 | 0 | 0 | 0 | Southern |
| Williamson | 8 | 36 | 154 | 3 | 0 | 0 | 0 | Southern |
